# VSIG4(+) peritoneal macrophages induce apoptosis of double-positive thymocyte via the secretion of TNF-α in a CLP-induced sepsis model resulting in thymic atrophy

**DOI:** 10.1038/s41419-021-03806-5

**Published:** 2021-05-22

**Authors:** Hae-Yun Cho, Yun Gyeong Yang, Youkyoung Jeon, Chae-Kwan Lee, InHak Choi, Soo-Woong Lee

**Affiliations:** 1 Innovative Therapeutics Research Institute, College of Medicine, Inje University, Busan, Republic of Korea; 2 Department of Microbiology and Immunology, Inje University College of Medicine, Busan, Republic of Korea; 3 Department of Occupational and Environmental Medicine, College of Medical & Institute of Environmental and Occupational Medicine, Inje University, Busan, Republic of Korea; 4 Department of Convergence Biomedical Science, College of Medicine, Inje University, Busan, Republic of Korea

**Keywords:** Imaging the immune system, Bacterial infection

## Abstract

Thymic atrophy in sepsis is a critical disadvantage because it induces immunosuppression and increases the mortality rate as the disease progresses. However, the exact mechanism of thymic atrophy has not been fully elucidated. In this study, we discovered a novel role for VSIG4-positive peritoneal macrophages (V4(+) cells) as the principal cells that induce thymic atrophy and thymocyte apoptosis. In CLP-induced mice, V4(+) cells were activated after ingestion of invading microbes, and the majority of these cells migrated into the thymus. Furthermore, these cells underwent a phenotypic shift from V4(+) to V4(−) and from MHC II(low) to MHC II(+). In coculture with thymocytes, V4(+) cells mainly induced apoptosis in DP thymocytes via the secretion of TNF-α. However, there was little effect on CD4 or CD8 SP and DN thymocytes. V4(−) cells showed low levels of activity compared to V4(+) cells. Thymic atrophy in CLP-induced V4(KO) mice was much less severe than that in CLP-induced wild-type mice. In addition, V4(KO) peritoneal macrophages also showed similar activity to V4(−) cells. Taken together, the current study demonstrates that V4(+) cells play important roles in inducing immunosuppression via thymic atrophy in the context of severe infection. These data also suggest that controlling the function of V4(+) cells may play a crucial role in the development of new therapies to prevent thymocyte apoptosis in sepsis.

## Introduction

Sepsis is a major cause of death and is associated with high rates of mortality and morbidity worldwide^1^. Approximately 750,000 cases are reported in the United States annually, and the mortality rate is as high as 28–50%^2^. Sepsis occurs as an early inflammatory response to infection, and patients with sepsis are often immunosuppressed. Extensive and profound immune cell apoptosis can be induced in multiple experimental animal models and has been shown in human studies^3,4^. Increased lymphocyte apoptosis correlates with immunosuppression, indicating that the immune system can fail to eliminate primary infections and is vulnerable to progressing to secondary nosocomial infections^5^. Because this event has been shown to be associated with mortality, several investigators have tried to inhibit multiple points along the intrinsic or extrinsic apoptotic pathways to improve survival in animal studies^3,6,7^. However, the reason for the different survival rates has yet to be elucidated.

The thymus is the primary lymphoid organ in which bone marrow-derived T-cell precursors develop into mature T cells. Thymocyte maturation depends on essential signals produced by dendritic cells, macrophages, endothelial cells, fibroblasts, and thymic epithelial cells that surround immature thymocytes^8^. Thymocyte fate during differentiation is decided according to interactions with self-peptide ligands presented on nonlymphoid thymic cells^9^. Most thymocytes that receive a strong TCR signal undergo activation-induced apoptosis. However, other cells that receive an intermediate-strength TCR signal survive and differentiate into CD4^+^ or CD8^+^ single-positive cells^10^. Under certain conditions, such as aging, hormone fluctuations, and infection, the thymus can undergo a specific state, such as involution, indicating the apoptotic death of many thymocytes^11^. Acute thymic atrophy has also been observed in sepsis and is characterized by the specific loss of CD4^+^CD8^+^ double-positive thymocytes, thus resulting in a dramatic reduction in T-cell development and immunosuppression^12,13^. It is well known that thymic atrophy is caused by microbial infection-induced sepsis, but the exact mechanism is unclear.

The V-set and Ig domain-containing 4 (VSIG4) protein is a member of the B7 family that inhibits T-cell activation and proliferation. Vogt et al.^14^ noted that VSIG4 was expressed on resting peritoneal macrophages but was downregulated upon macrophage activation. The researchers also showed that VSIG4 was a negative regulator of T-cell proliferation and was downregulated in autoimmune tissues. In humans, VSIG4 is also known as the complement receptor of the immunoglobulin superfamily (CRIg)^15^. Thus, it is thought that VSIG4 (CRIg)-positive macrophages play a significant role in maintaining homeostasis by increasing complement-dependent phagocytosis in mice and humans^16^. VSIG4 is expressed on subsets of tissue-resident macrophages, such as Kupffer cells in the liver, and acts as a critical player in pathogen recognition and clearance^17^. However, the role of VSIG4-expressing macrophages in sepsis has not been elucidated.

In our pilot study, we carried out a microarray analysis of circulating immune cells from the cecal ligation and puncture (CLP)-induced mouse model of abdominal sepsis and found increased gene expression of VSIG4 among several others. The finding encouraged us to carry out further in-depth study on the pathogenic role of VSIG4-positive peritoneal macrophages in a CLP-induced sepsis model using wild-type and VSIG4-knockout mice. The results support strongly that VSIG4 positive macrophages are responsible for double-positive thymocyte apoptosis and thymic atrophy occurring in the sepsis, via migration from the peritoneal cavity into thymus.

## Materials and methods

### Animals

Female C57BL/6 mice at 8–10 wk of age (18–22 g) were purchased from Orient Bio (Seognam, Korea). *VSIG4* KO mice with a C57/B6 background (VSIG4KO) were generously provided by Dr. Campagne (Genentech). The animals were kept in isolated cages with a 12-h light-dark schedule and fed with standard food pellets and water. All experimental protocols were approved by the Institutional Animal Care and Use Committee at Inje University College of Medicine.

### Antibodies

The following antibodies were purchased from eBioscience (San Diego, CA) for flow cytometry: fluorescein isothiocyanate (FITC)-conjugated antibodies against CD11b (M1/70), CD4 (Gk1.50), and Rat IgG2b isotype control; phycoerythrin (PE)-conjugated antibodies against TLR4 (MTS510), RP105 (RP/14), CD80 (16-10A1), CD86 (GL1), MHC class I (AF6-88.5.5.3), MHC class II (M5/114.15.2), CD11b (M1/70), and isotype control; allophycocyanin (APC)-conjugated antibodies against CD8 (Ly-2), VSIG4 (NLA14), and isotype control; and purified anti-CD16/32 (2.4G2). Functional grade anti-TNF-α (MP6-XT22) was purchased from eBioscience for T-cell activation. 7-AAD was purchased from eBioscience and PE-conjugated Annexin V was purchase from BD Bioscience (Franklin Lakes, NJ) for apoptosis.

### Cecal ligation and puncture

Sepsis was induced in mice using the cecal ligation and puncture (CLP) method as defined by Baker et al.^18^. Mice were randomly divided into two groups (*n* = 6 per group): Sham-operated and CLP-induced mice. Mice were anesthetized using the combination of ketamine (HUONS) and xylazine (Bayer Korea) intraperitoneally. The abdomen was disinfected with povidone iodine swabsticks, and a 1.5 cm midline abdominal incision was conducted. The cecum was exposed, ligated below the ileocecal valve with a 3-0 silk, and then punctured with a 26-gauge needle for the sepsis induction. After the operation, the cecum was placed back to the original location. Only abdominal incision was performed on the WT control mice, exposed for 5 min, and then closed in layers. All animals (Sham and CLP) received fluid resuscitation with 0.5 ml saline (given subcutaneously in the nuchal region). Pain control in both groups was achieved by treatment with 0.05 mg/kg buprenorphine every 12 h. The mice were returned to their cages with heating pads until full recovery.

### Imaging of mice

Peritoneal macrophages or VSIG4(+) and VSIG4(−) cells were stained with VivoTrack 680 (PerkinElmer) at RT for 15 min and washed 3 times with PBS. CLP-induced mice were randomly divided into two groups (*n* = 6 per group): VSIG4(+) and VSIG4(−), and stained cells were injected into peritoneal cavity of each mice and imaged 24 h later. For image detection, all mice were anesthetized with the combination of ketamine and xylazine intraperitoneally or sacrificed before imaging. The hair on the abdominal region of the mice was removed using a safety razor. Whole body or organs removed mouse was quantified by measuring the near-infrared fluorescence (excitation: 670 ± 15; emission: 700 ± 15) using Xenogen IVIS^TM^ and Living Image Software (Xenogen). The thymus were also harvested individually and imaged with IVIS.

### Separation of VSIG4(+) cells, preparation of thymocytes

Mouse primary immune cells were positively separated by high-gradient magnetic sorting using the magnetic cell sorting (MACS) and MACS techniques according to the protocol provided by the manufacturer (Miltenyi Biotec, Bergisch Gladbach, Germany). VSIG4(+) cells were enriched from three C57BL/6 peritoneal cells using anti-VISG4 and magnetic beads (Miltenyi Biotec). The cells were separated using MACS columns and yielded approximately >95% pure cells. VSIG4(−) cells were enriched from peritoneal cells without VSIG4(+) cells, using anti-CD11b antibody-conjugated beads. For prepared thymocytes, freshly isolated thymus from normal mice were placed in cold phosphate-buffered saline (PBS), and immediately homogenized. Total cells were filtered through cell strainer to remove tissue debris. Thymocyte suspensions were used for all experiments.

### Cytokine measurements

Cells were cultured for various times in medium, and the supernatants were then collected and clarified by centrifugation. The level of TNF-α was measured using mouse ELISA MAX Deluxe kit from BioLegend (San Diego, CA) according to the manufacturer’s instructions. Data were analyzed using the standard curve plotted for this purpose to calculate the quantity of TNF-α.

### Flow cytometry analysis

For the expression of VSIG4, mouse primary immune cells were isolated from peritoneal cavity, thymus, and spleen. Cells were washed with FACS buffer (PBS containing 1% FCS and 0.05% NaN_3_), incubated with FcR blocker (2.4G2) at 4 °C for 10 min to block nonspecific antibody binding, and stained with anti-mouse VSIG4 antibody at 4 °C for 20 min. Cells were analyzed using BD FACSCanto II (BD Bioscience, Franklin Lakes, NJ), and data were analyzed using FlowJo software. Protein expression was represented as mean fluorescence intensity (MFI). For apoptosis analysis, cells were washed with PBS and binding buffer. Cells were stained with annexin V and 7-AAD, and then analyzed by flow cytometry.

### Phagocytosis assays

Phagocytosis assay was performed using peritoneal macrophages from wild-type and VSIG4 knock-out mouse. VSIG4(+) and VSIG4(−) cells were isolated and incubated with 1 mg/ml FITC-dextran (Mr = 2,000,000; Sigma) for 30 min in serum-free RPMI at 37 °C, 5% CO_2_. Cells were then washed three times with cold PBS to remove fluorescent beads that had not been internalized. Finally, cells were scraped from the plate, stained with PE-conjugated CD11b antibody, and analyzed by flow cytometry. For phagocytosis, thymocyte were stained with calcein-AM (final concentration 1 μM, Invitrogen) and cocultured with VSIG4(+) and VSIG4(−) cells for 6 h. And then cells were stained with anti-CD11b antibody and analyzed by flow cytometry

### Statistical analysis

All experiments are randomized and statistical analysis was performed with GraphPad Prism 5.0 software (San Diego, CA, USA). Data were expressed as mean ± S.E.M. of the indicated number of experiments. The statistical significance of data was estimated using a one-way ANOVA for unpaired observations, and the *p* values < 0.05 was considered statistically significant.

## Results

### Peritoneal macrophages were found in the thymus after CLP induction

**Fig. 1:**
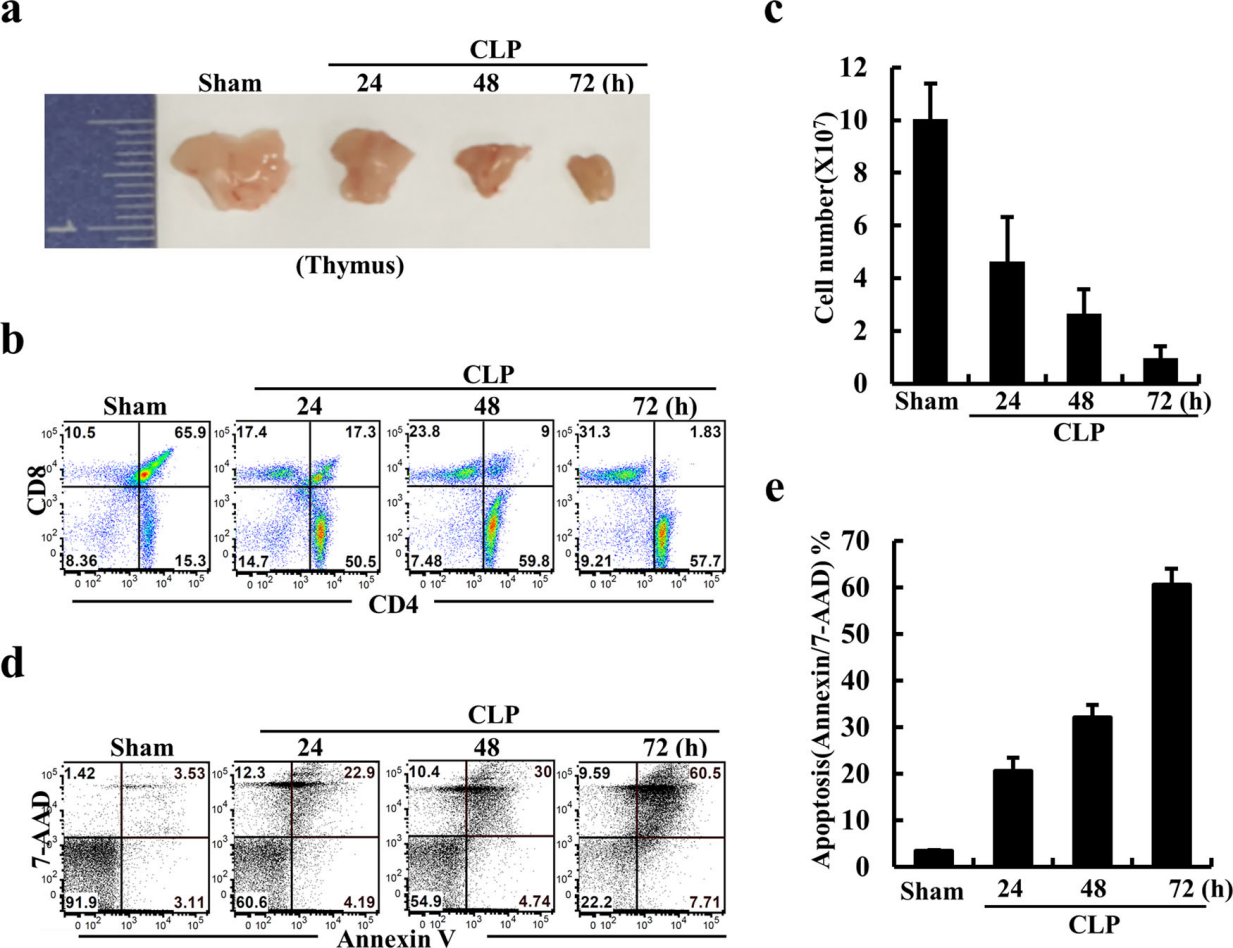
Analysis of thymus from CLP mice. **a** Thymus were isolated from sham- and CLP-induced mice at the indicated time points. **b** Total thymocytes were isolated from thymus prepared in (**a**). Cells were stained with CD4 and CD8 antibodies, and analyzed by flow cytometry. **c** Total thymocytes prepared in (**a**) were counted. **d** To detect apoptosis, total thymocytes prepared in (**a**) were stained with Annexin V and 7AAD, and analyzed by flow cytometry. **e** The data indicates the percentage of apoptotic cells. The data represent the mean ± S.E.M. of three separate experiments. Sham mice indicate sham-operated mice and CLP mice indicate CLP-induced mice.

**Fig. 2:**
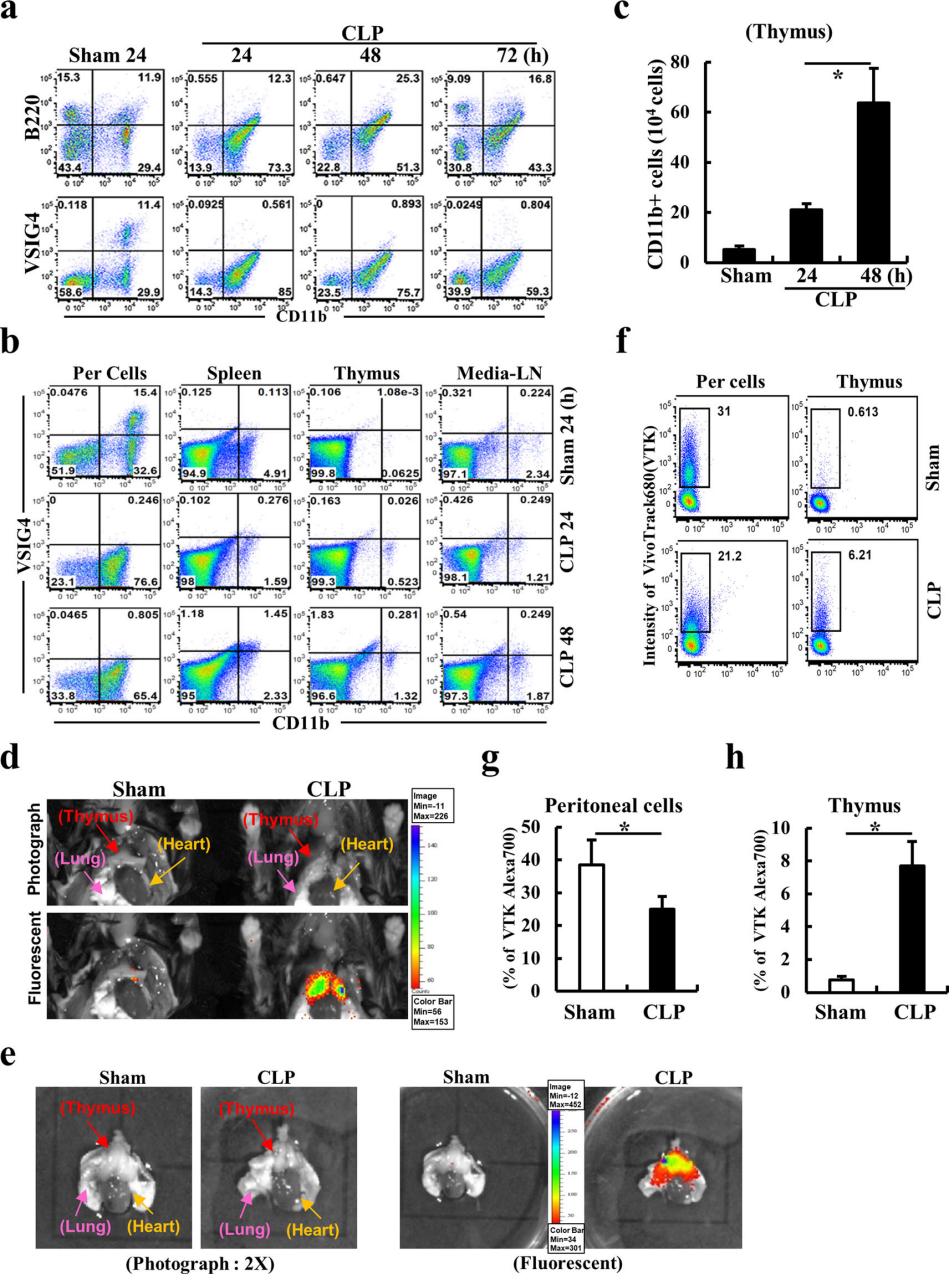
Analysis on the migration of peritoneal macrophages after CLP induction. **a** Total peritoneal cells were isolated from sham and CLP mice. Cells were stained with anti-B220 and anti-CD11b antibodies or anti-VSIG4 and anti-CD11b antibodies, and analyzed by flow cytometry. **b** CLP was induced in mice and after 24 or 48 h, immune cells were isolated from the peritoneal cavity, spleen, thymus, and mediastinal lymph nodes. Cells from sham mice were used as control. Cells were stained with VSIG4 and CD11b antibodies, and analyzed by flow cytometry. **c** CD11b^+^ cells in thymus from sham and CLP mice (24 and 48 h) were counted. **d** Peritoneal macrophages were isolated and stained with VivoTracker, and then injected into the peritoneal cavities of sham (left) or CLP mice (right). After 24 h, the mice were euthanized, and the peritoneal cavity was washed with PBS several times, and then analyzed with an In Vivo Imaging System (IVIS Spectrum). **e** Organs containing thymus were isolated from sham or CLP mice and analyzed with an in vivo imaging system (IVIS Spectrum). **f** Immune cells were isolated from the peritoneal cavity and thymus of mice as shown in (**d**), and the fluorescence intensity of the cells was analyzed by flow cytometry. **g** Number of VivoTracker positive cells from peritoneal cavity and **h** thymus as shown in (**e**) were counted using Alexa 700. The data represent the mean ± S.E.M. of three separate experiments. *p < 0.05, ANOVA. Per Cells indicate peritoneal cells, and Media-LN indicates the mediastinal lymph node around the thymus.

As known previously, when sepsis was induced by CLP in mice, we also found that the thymus becomes atrophy as sepsis progresses (Fig. 1a). Furthermore, the CD4/CD8 double-positive (DP) thymocytes disappeared significantly in a time-dependent manner (CLP/Sham: 27.4% at CLP 24 h, 13.6% at CLP 48 h and 3.5% at CLP 72 h) (Fig. 1b). As a result, the number of thymocytes decreased rapidly (CLP/Sham: 46% after 24 h, 27% after 48 h, and 8.6% after 72 h) (Fig. 1c) and the number of dead cells was increased in a time-dependent manner (CLP/Sham: 6.5-fold at 24 h, 7.5-fold at 48 h, and 13-fold at 72 h) (Fig. 1d, e). Since we were curious about the putative roles of VSIG4 positive macrophages (V4(+)) in the CLP mice, we studied the cells in the peritoneal immune cells using flow cytometry. In normal mice, V4(+) cells were approximately one-third of total peritoneal macrophages (Fig. 2a). In the CLP mice, to our surprise, most of them disappeared from the peritoneal cavity and a different type of macrophages found there (Fig. 2a). As we presumed that original peritoneal macrophages might have migrated from peritoneal cavity to another organ(s) after CLP induction, immune cells from the peritoneal cavity, spleen, thymus, and thymus-bound lymph nodes (Media-LN) in sham and CLP mice were analyzed. Unexpectedly, none of the organs showed significant difference in the number of V4(+) cells between the sham and CLP mice (Fig. 2b). Nevertheless, we found that the number of macrophages significantly increased in thymus after CLP induction compared to that of sham mice (CLP/Sham: 4.1-fold (2.1 × 10^5^/0.51 × 10^5^) after 24 h, 12.5-fold (6.4 × 10^5^/0.51 × 10^5^) after 48 h) (Fig. 2b, c).

As an alternative approach to trace the disappeared peritoneal macrophages, we employed an In Vivo Image Analyzer (IVIA). Peritoneal macrophages were first isolated from normal mice and stained with VivoTracker. The stained cells were injected into the peritoneal cavity of sham or CLP mice, which were analyzed after 24 h using IVIA. We could observe a clear distinction between the sham and CLP mice in the thymus. In contrast to the sham mice, the CLP mice exhibited strong VivoTracker signal in the thymus (Fig. 2d). When organs were isolated from body, we found that CLP mice underwent severe thymic atrophy compared to sham mice (Fig. 2e, left). Since the signal was maintained active in the thymus even after being isolated from the body (Fig. 2e, right), the immune cells isolated from the peritoneal cavity and thymus were subjected to flow cytometry. The quantitative differences in VivoTracker cells were clearly seen between the organs and between the sham and CLP mice (Fig. 2f). In the sham mice, VivoTracker positive cells observed in the thymus were <2% (0.613/31) of those in the peritoneal cavity, while more than 29% (6.21/21.2) in the CLP mice (Fig. 2g). The finding of VivoTracker positive cells in the thymus of sham mice may be significant in a point that it indicates potential migration of peritoneal macrophages to thymus under physiological conditions, which increases more than 10-fold (6.21/0.613) upon CLP induction (Fig. 2h). Taken together, the above results suggested strongly that peritoneal macrophages may migrate from the peritoneal cavity to thymus upon CLP induction in mice.

### V4(+)/MHC II(low) cells change to V4(−)/MHC II (+) cells in the thymus after CLP induction

**Fig. 3:**
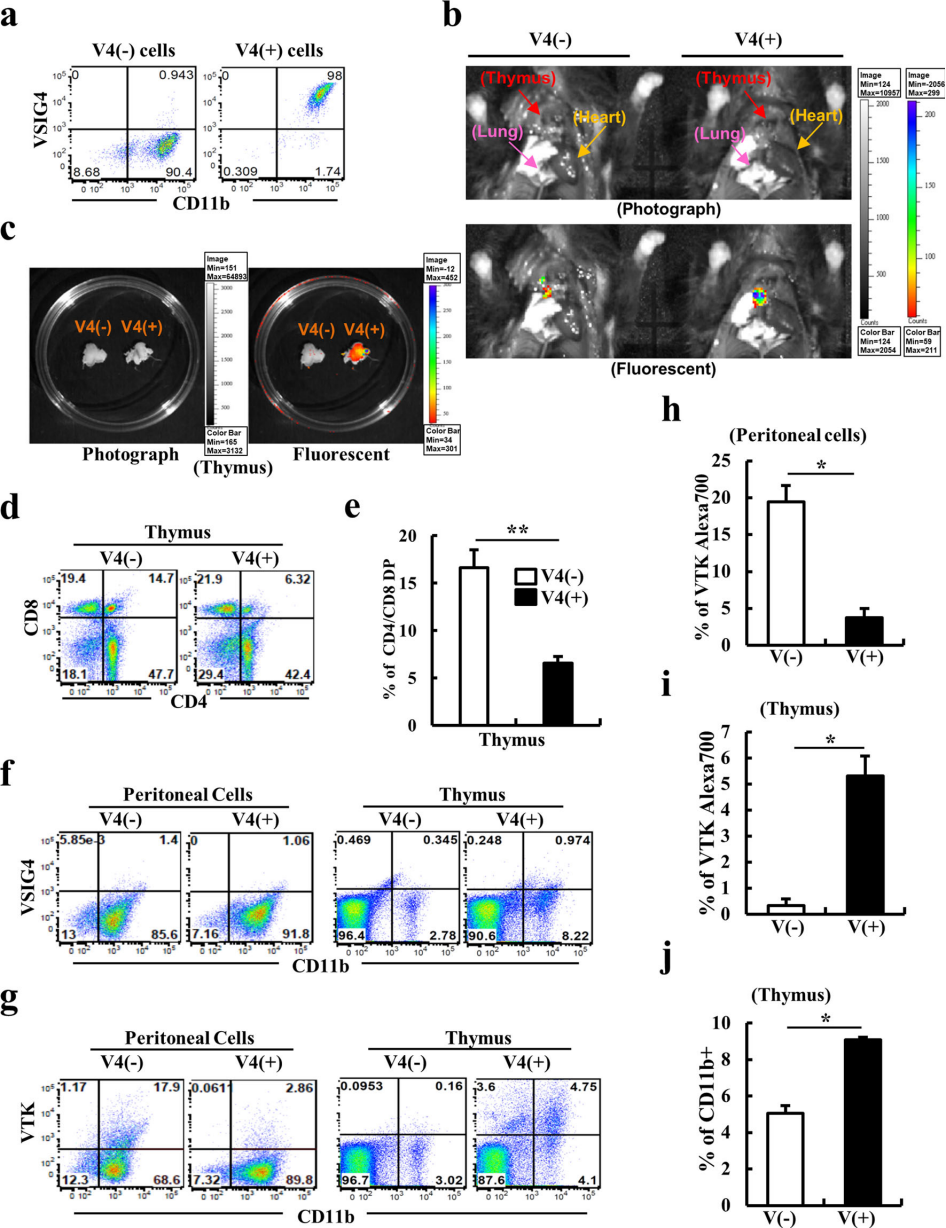
In vivo image analysis of purified V4(+) and V4(−) cells. **a** Peritoneal cells were isolated from wild-type mice, and V4(+) and V4(−) cells were purified using anti-VSIG4 or anti-CD11b antibody and MACS MicroBeads. Cells purity >90% was obtained. **b** V4(+) and V4(−) cells were stained with VivoTracker, and the stained cells were injected into the peritoneal cavities of CLP mice. After 24 h, the mice were euthanized, and the peritoneal cavity was washed with PBS three times. Then, the fluorescence intensities of different organs were detected with an In Vivo Imaging System. **c** The thymus was only isolated from V4(−) cell-injected mice, but the thymus from V4(+) cell-injected mice showed severe contraction, so it was isolated with the surrounding tissue and analyzed with an IVIS. **d** After IVIS analysis, thymocytes were purified, and cells were stained with CD4 and CD8 antibodies, and analyzed by flow cytometry. **e** The data indicates the percentage of CD4/CD8 DP cells. **f** After IVIS analysis, immune cells isolated from the peritoneal cavity and thymus were stained with CD11b and VSIG4 antibodies, and analyzed by flow cytometry. **g** Peritoneal cells and thymocytes as shown in (**b**) were isolated and analyzed with an IVIS. **h** Number of VivoTracker positive cells from peritoneal cavity and **i** thymus as shown in (**b**) were counted using Alexa 700. **j** CD11b^+^ cells from thymus as shown in (**f**) were counted. The data represent the mean ± S.E.M. of three separate experiments. *p < 0.05, **p < 0.01, ANOVA. V4 indicates VSIG4, and VTK indicates the activity of VivoTracker.

Although peritoneal macrophages were shown to be present in the thymus after CLP induction, there remained a question if they were either V4(+) or V4(−) cells, because V4(+) cells were not found in the thymus. In order to answer the question, we purified the V4(+) and V4(−) cells separately from the peritoneal immune cells (Fig. 3a) and repeated IVIA analysis using them. The purified V4(+) and V4(−) cells were stained with VivoTracker and then injected separately into the peritoneal cavity of CLP mice. The VivoTracker signal was higher in the thymus of the V4(+) cell-injected mice than that of V4(−) cell-injected mice (Fig. 3b, c). The presence of weak VivoTracker signals in the thymus of V4(−) cell-injected mice might be due to the contaminating V4(+) cells, which were not completely removed during the cell separation process. When the thymus was further analyzed, the number of DP thymocytes was greatly decreased in the V4(+) cell-injected mice than that of V4(−) cell-injected mice (V4(+)/V4(−): 42% (7.43/17.7), V4(−)/sham: 25.4% (17.7/ 69.6), V4(+)/sham: 10.7% (7.43/ 69.6)) (Fig. 3d, e).

For a more detailed analysis of the putative cell migration, macrophages were isolated from peritoneal cavity and thymus of the V4(+) cell- and V4(−) cell-injected mice, and analyzed using flow cytometry. When analyzed cells in peritoneal cavity, only V4(−) macrophages were found in the V4(+) cell- and V4(−) cell-injected mice after CLP-induction (Fig. 3f). Also, VivoTracker positive and negative macrophages were found in peritoneal cavity of the V4(−) cell-injected mice, while VivoTracker negative macrophages were found mainly in the V4(+) cell-injected mice (Fig. 3g). VivoTracker positive macrophages were reduced (V4(−) cell-injected mice: 19%, V4(+) cell-injected mice: 3.7%) (Fig. 3h), and VivoTracker negative new macrophages were significantly increased in peritoneal cavity after CLP induction (Fig. 3g).

In the thymus, abundant macrophages from V4(+) cell-injected mice was indicated by V4(−) (Fig. 3f) and the high intensity of the VivoTracker signal (VTK(+) cells/total cells: 5.24%) (Fig. 3g, i). In contrast, very few VivoTracker positive macrophages were only found in thymus from V4(−) cell-injected mice (VTK(+) cells/total cells: 0.22%) (Fig. 3g, i). VivoTracker negative macrophages in thymus might be a new macrophage which found in peritoneal cavity after CLP induction (Fig. 3g). Because it is known that the activated V4(+) cells changes to V4(−) cells^14^, we further analyzed these cells using VSIG4 and CD11b antibodies. As predicted, most of the cells were identified as V4(−) cells in thymus (Fig. 3f, j). These results demonstrate that among peritoneal macrophages, V4(+) cells mainly migrate to the thymus after CLP. We presume that, while in the process of migration after ingestion of microbes, cells may change to V4(−) cells.

**Fig. 4:**
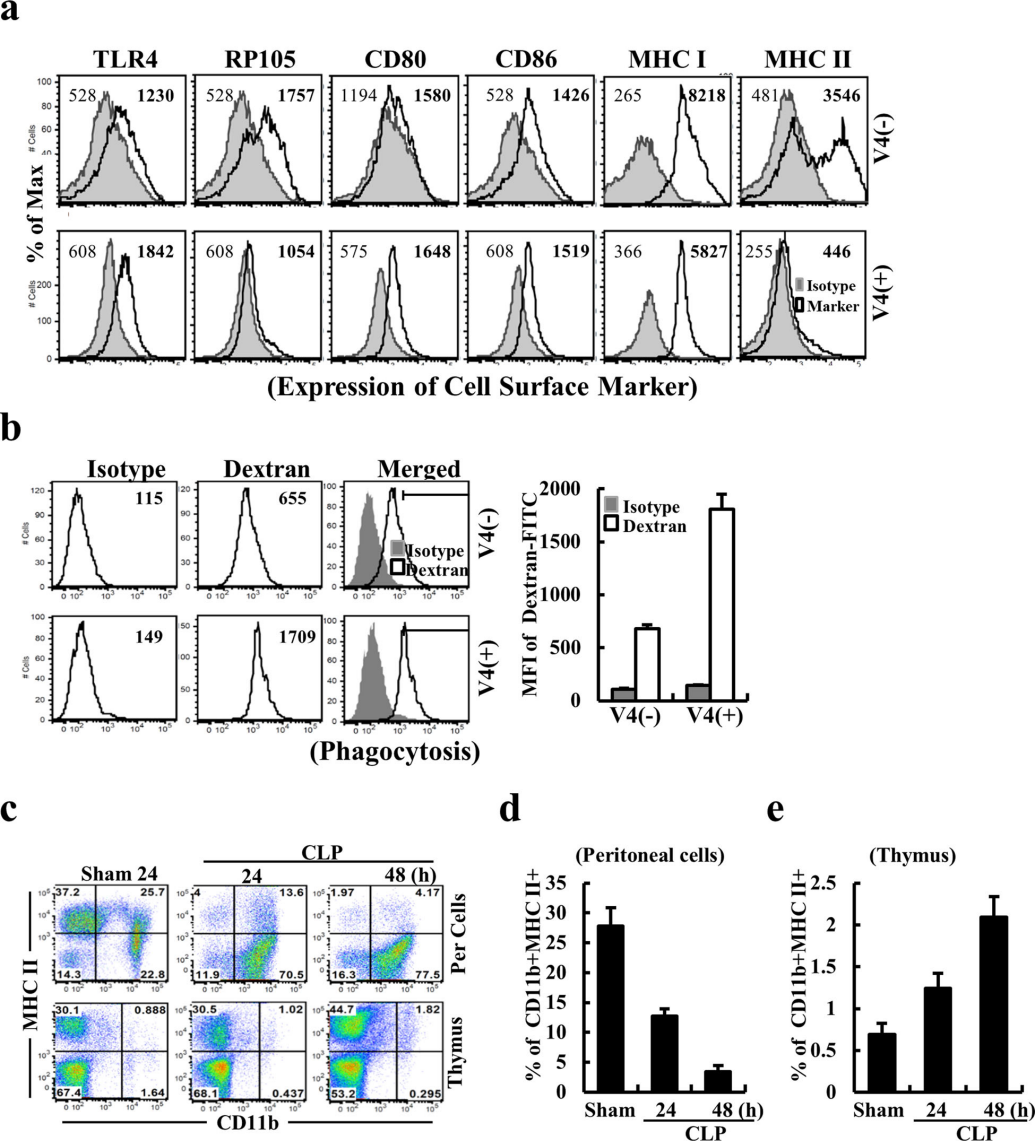
Analysis of the phenotype, phagocytosis, and migration of V4(+) cells. **a** Peritoneal cells were isolated from wild-type mice, and V4(+) and V4(−) cells were purified using anti-VSIG4 antibody and MACS MicroBeads. Cells were stained with TLR4, RP105, CD80, CD86, MHC I, and MHC II antibodies, and analyzed by flow cytometry. The data represent the average value of triplicates. **b** V4(+) and V4(−) cells were cultured with dextran for 30 min, and phagocytic cells were analyzed by flow cytometry. The data represent the average value of triplicates. **c** Purified V4(+) cells were injected into peritoneal cavity of sham or CLP mice. Peritoneal cells and thymocytes were isolated from sham or CLP mice for 24 and 48 h. Cells were stained with CD11b and MHC II antibodies, and analyzed by flow cytometry. **d** Number of CD11b(+)/MHC II(+) cells from peritoneal cavity and **e** thymus as shown in (**c**) were counted. The data represent the average value of triplicates.

Because V4(+) and V4(−) cells showed different migratory behaviors under CLP conditions, their phenotypes and functions were further analyzed. When cells were stained with various cell surface markers, V4(+) cells showed high expression levels of TLR4, CD80, CD86, and MHC I, but low expression levels of RP105 and MHC II. In contrast, V4(−) cells showed high expression levels of them except CD80 (Fig. 4a). When phagocytosis was analyzed in vitro using dextran, V4(+) cells exhibited much higher capacity than V4(−) (V4(−) cells: 655, V4(+) cells: 1709) (Fig. 4b). Furthermore, as shown in Fig. 4c, the number of macrophages in the thymus was significantly increased after CLP induction in a time-dependent manner, and these cells exhibited MHC II(+) phenotype (Fig. 4e). While the number of MHC II(+) macrophages in peritoneal cavity was decreased in a time-dependent manner (Fig. 4d). The result supported that V4(+) cells change their phenotype from MHC II(low) to MHC II(+) after CLP induction, suggesting some functional roles of them such as phagocytosis in thymus into where they migrated.

### V4(+) cells induce thymocyte apoptosis via TNF-α release

**Fig. 5:**
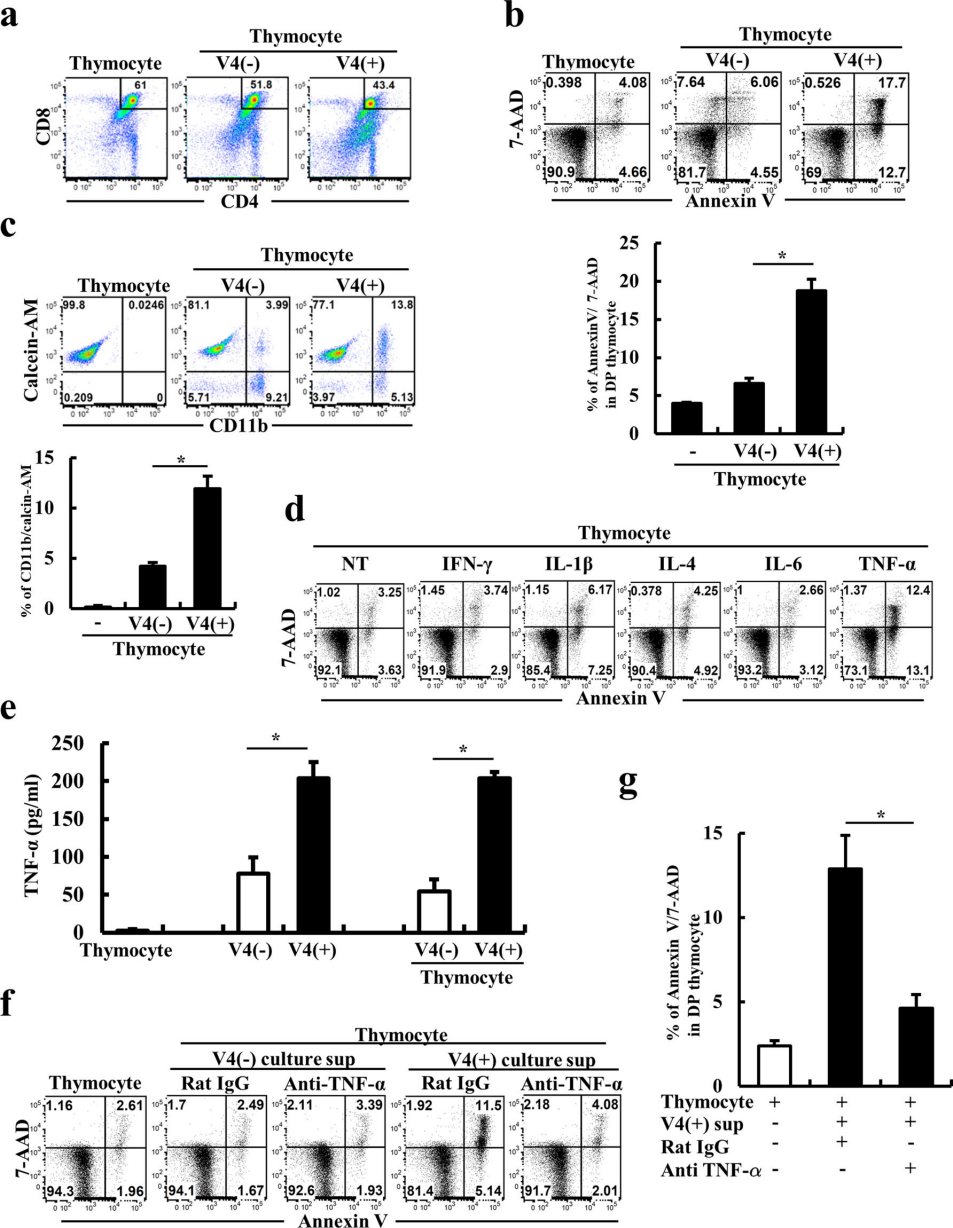
V4(+) cells induce thymocyte apoptosis and phagocytosis. **a** Peritoneal cells were isolated from wild-type mice, and V4(+) and V4(−) cells were purified using anti-VSIG4 antibody or anti-CD11b antibody and MACS MicroBeads. Thymocytes were isolated from thymuses obtained from wild-type mice, and were cocultured with purified V4(+) or V4(−) cells for 17 h. After coculture, the cells were stained with CD4 or CD8 antibodies, and analyzed by flow cytometry. **b** Annexin V and 7-AAD-stained DP thymocytes were gated, and apoptosis was analyzed by flow cytometry. **c** Thymocytes were stained with calcein-AM, and cocultured with V4(+) or V4(−) cells for 6 h. After culture, the cells were stained for CD11b, and calcein-AM activity was analyzed by flow cytometry. **d** Thymocytes were cultured with various cytokines, such as IFN-γ, IL-1β, IL-4, IL-6, and TNF-α. After 24 h, the cells were stained with Annexin V and 7-AAD, and apoptosis in the gated DP thymocytes was analyzed by flow cytometry. The data represent the average value of triplicates. **e** V4(+) or V4(−) cells were cocultured with thymocytes for 17 h, and then the concentration of TNF-α in the culture supernatant was analyzed using an ELISA kit. **f** The culture supernatants prepared from (**e**) were mixed with rat IgG as a control or anti-TNF-α antibody to block TNF-α, and added to the thymocyte culture. After 17 h, the cells were stained with Annexin V and 7-AAD, and apoptosis in the gated DP thymocytes was analyzed by flow cytometry. **g** Apoptotic cells in the gated DP thymocytes were counted. The data represent the mean ± S.E.M. of three separate experiments. *p < 0.05, ANOVA.

In order to see the effects of V4(+) cells in the thymus, thymocytes were isolated from normal mice and cocultured with purified V4(+) or V4(−) cells in vitro. When cocultured cells were analyzed, V4(+) cells induced apoptosis in many DP thymocytes (V4(+)/V4(−): 2.9-fold), but hardly affected CD4 or CD8 single-positive cells (Fig. 5a, b). In contrast, the V4(−) cells had little effect on thymocyte viability. When cells were cocultured with Calcein-AM-stained thymocytes, V4(+) cells showed higher levels of phagocytosis than V4(−) cells (V4(+)/V4(−): 3.5-fold) (Fig. 5c).

We further examined putative soluble factors of V4(+) cells affecting the viability of thymocytes. When cultured with various cytokines, such as INF-γ, IL-1β, IL-4, IL-6, and TNF-α, DP thymocytes underwent apoptosis exclusively in the presence of TNF-α (TNF-α/NT: 3.8-fold) (Fig. 5d). As shown in Fig. 5e, V4(+) cells surprisingly secreted higher levels of TNF-α than V4(−) cells when cultured with or without thymocytes (V4(+)/V4(−): 2.9-fold). When thymocytes were treated with the culture supernatants of V4(+) or V4(−) cells, and with the coculture supernatants of V4(+) or V4(−) cells with thymocytes, apoptosis of DP thymocytes was increased 1.9-fold by the supernatant of V4(+) cells, whereas this effect was decreased by TNF-α blocking antibody (Anti-TNF-α/Rat IgG: 69%) (Fig. 5f, g). These results indicate that in CLP induction, V4(+) cells may induce apoptosis of DP thymocyte via the secretion of TNF-α.

### Functional study in CLP-induced VSIG4-knockout mice

**Fig. 6:**
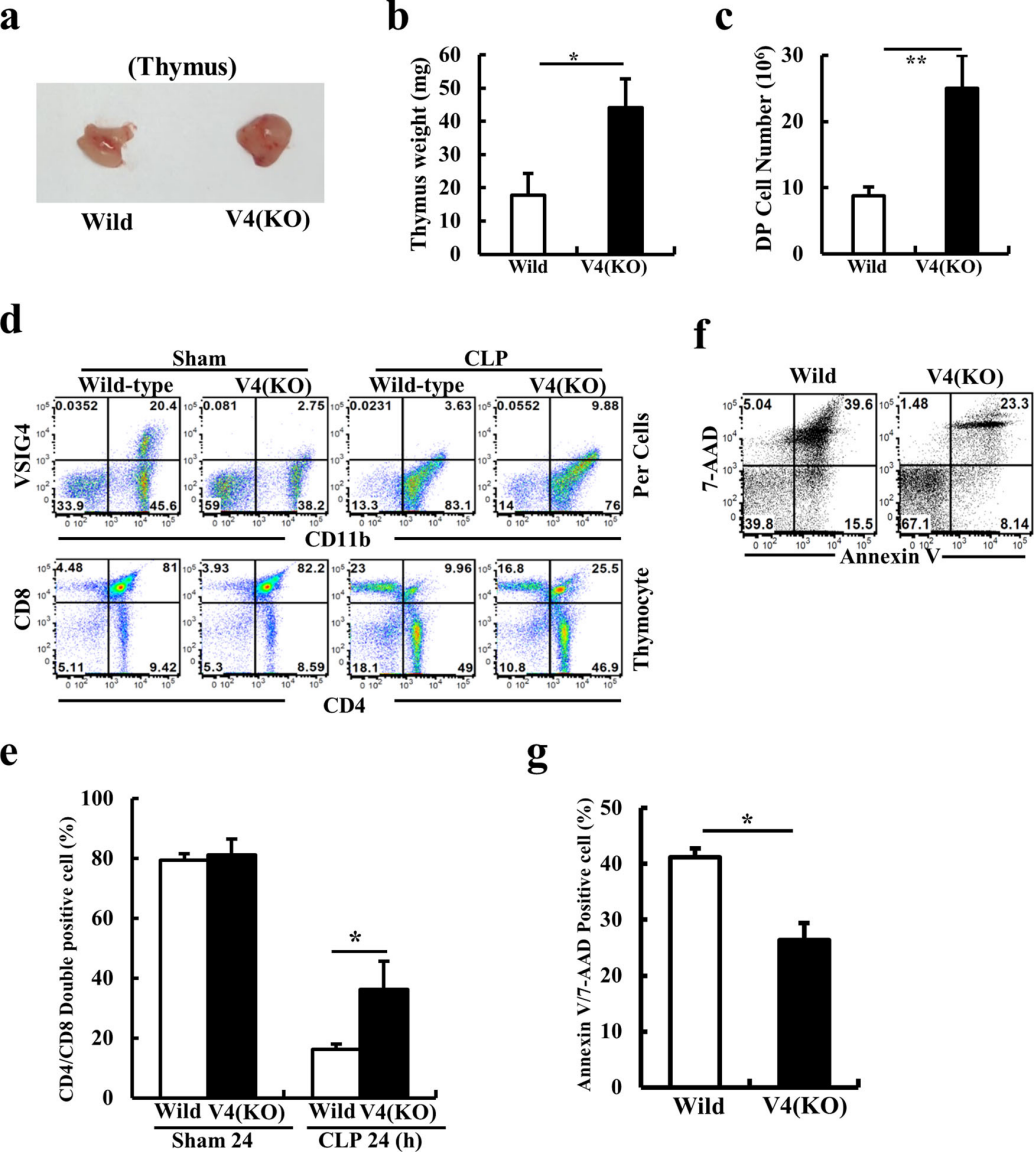
Study using CLP-induced V4(KO) mice. **a** Wild-type and V4(KO) mice underwent CLP induction for 24 h. The structure of the thymus was analyzed, and **b** thymocyte weight and **c** total cell number was estimated. **d** Cells were isolated from the peritoneal cavity and thymus as shown in (**a**), and stained with anti-VSIG4 and anti-CD11b antibodies to label peritoneal cells or anti-CD4 and anti-CD8 antibodies to label thymocytes. Then, the cells were analyzed by flow cytometry. **e** The percentage of DP thymocytes was estimated in total thymocytes from CLP-induced wild-type and V4(KO) mice. **f** Thymocytes from CLP-induced wild-type and V4(KO) mice cells were stained with 7-AAD and Annexin V. Thymocyte apoptosis was analyzed by flow cytometry. **g** Apoptotic cells in thymocytes were counted. The data represent the mean ± S.E.M. of three separate experiments. *p < 0.05, **p < 0.01, ANOVA. V4(KO) indicates VSIG4-knockout mice and sham indicates sham-operated mice.

To further confirm our new findings that V4(+) cells induce thymic atrophy upon CLP induction, we examined the thymus atrophy of the VSIG4-knockout (V4(KO)) mice. When the purified V4(+) and V4(KO) cells stained with VivoTracker were injected into peritoneal cavity of normal mice after CLP induction, V4(+) cells actively migrate to thymus, while few V4(KO) cells have been found after CLP induction (Supplementary Fig. 1a-d). As shown in Fig. 6a, both the size and structure of the thymus in V4(KO) mice were similar to those of the wild-type mice and did not change after CLP-induction, whereas the thymus in wild-type mice showed severe atrophy after CLP induction (Fig. 6b). 24 h after CLP induction, the number of DP thymocytes in the thymus of V4(KO) mice was decreased much than that of sham mice, but was 2.5-fold higher than that of wild-type mice (25.5/9.96) (Fig. 6c). The number of apoptotic cells in the thymus from V4(KO) mice was also much less than that in wild-type mice (64.5%: 26.7/41.4) (Fig. 6d, e). When thymocytes were stained with 7-AAD and Annexin V, apoptotic cells in wild-type mice were 1.8-fold higher than that of V4(KO) (Fig. 6f, g). Together, our data indicate that CLP–induced thymocyte apoptosis and thymic atrophy are less severe in V4(KO) mice then wild-type mice than wild-type mice, supporting the role of V4(+) cells in the process.

**Fig. 7:**
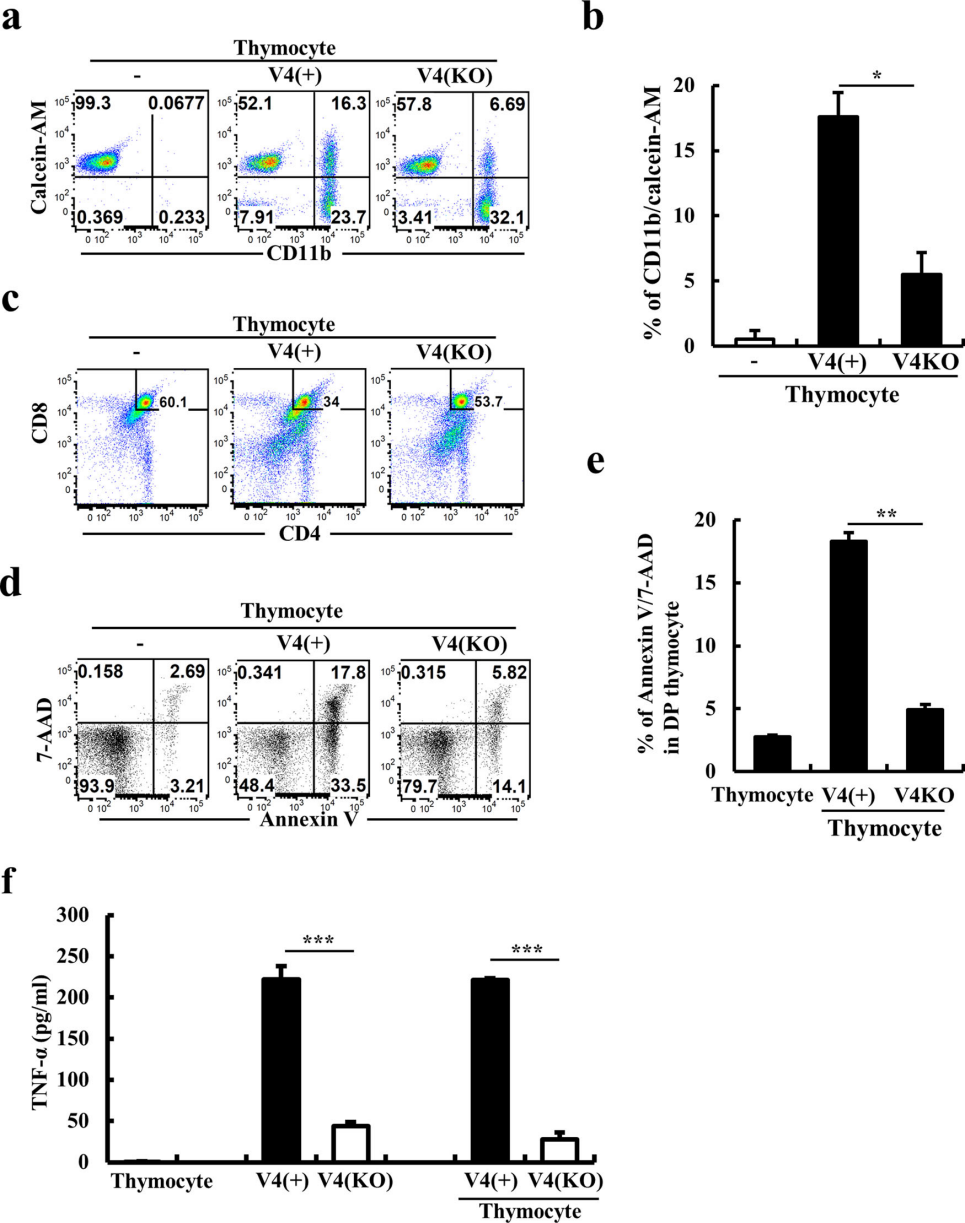
Functional study using V4(KO) macrophages. **a** V4(+) and V4(KO) macrophages were isolated from wild-type or V4(KO) mice using anti-VSIG4 or anti-CD11b antibody and MACS MicroBeads. Cells purity >95% was obtained. Thymocytes were stained with calcein-AM, and cocultured with purified V4(+) and V4(KO) cells for 6 h. After culture, the cells were stained for CD11b, and calcein-AM activity was analyzed by flow cytometry. **b** Fluorescence intensity of calcein-AM in cells was estimated by flow cytometry. **c** Thymocytes were cocultured with V4(+) or V4(KO) macrophages (2:1) for 17 h, and stained with anti-CD4 and anti-CD8 antibodies. Then, the cells were analyzed by flow cytometry. **d** The cells as shown in (**c**) were stained with Annexin V and 7-AAD, and apoptosis in the gated DP thymocytes was measured using flow cytometry. The data represent the average value of triplicates. **e** The cells as shown in (**d**) were stained with Annexin V and 7-AAD, and apoptosis in the gated DP thymocytes was analyzed by flow cytometry. **f** V4(+) and V4(KO) macrophages prepared in (**a**) were cultured with or without thymocyte for 17 h. The concentration of TNF-α in culture supernatants was measured. The data represent the mean ± S.E.M. of three separate experiments. *p < 0.05, ***p < 0.001, ANOVA.

Because V4(KO) mice were less affected than wild-type mice by CLP induction, we further evaluated the function of V4(KO) macrophages in vitro. When each cell was purified from total peritoneal cells of wild-type and V4(KO) mice using anti-VSIG4 and anti-CD11b antibody and cultured with Calcein-AM-stained thymocytes, V4(KO) macrophages exhibited less phagocytosis than that of wild-type V4(+) cells (Fig. 7a). The phagocytosis of wild-type V4(+) cells was 2.3-fold higher than that of V4(KO) cells (16.3/6.69) (Fig. 7b).

As shown in Fig. 7c, when purified V4(KO) macrophages were cocultured with wild-type thymocytes, the viability of DP thymocytes was less affected than when thymocytes were cocultured with wild-type V(+) cells (58.7%) (Fig. 7d, e). V4(KO) macrophages cultured with or without thymocytes secreted much less TNF-α than wild-type V4(+) cells (21.7%) (Fig. 7f). Taken together, our data indicate that immune activity in response to infection is less severe in V4(KO) mice than in wild-type mice, probably due to a reduction in the immune activity of V4(KO) macrophages. Therefore, blocking the function of V4(+) cells may be valuable to prevent thymic atrophy by severe infection.

## Discussion

Currently, the biggest challenge to the treatment of sepsis patients is that all clinical trials aiming to dampen the inflammatory response or targeting the cytokines failed^19^. For this reason, the discovery of new targets for treatment is critical and has been desperately demanded. But new targets for sepsis are still unclear. Our new findings in this study suggest strongly that V4(+) cells could be a potential target for sepsis treatment because they appear to cause sepsis-induced thymus atrophy. The thymus is an essential organ that produces T lymphocytes that respond to new antigens^20^. It is known as an early-stage organ damaged in sepsis^21,22^. Thymus damage would lead to an increased mortality due to immunodeficiency when new antigens, such as COVID-19, invade. Thus, in the early stages of sepsis treatment, the control of the VSIG4 positive macrophages is considered important to prevent thymus damage and to maintain patient immunity for the treatment process or/and to prevent recurrence after a treatment.

**Fig. 8:**
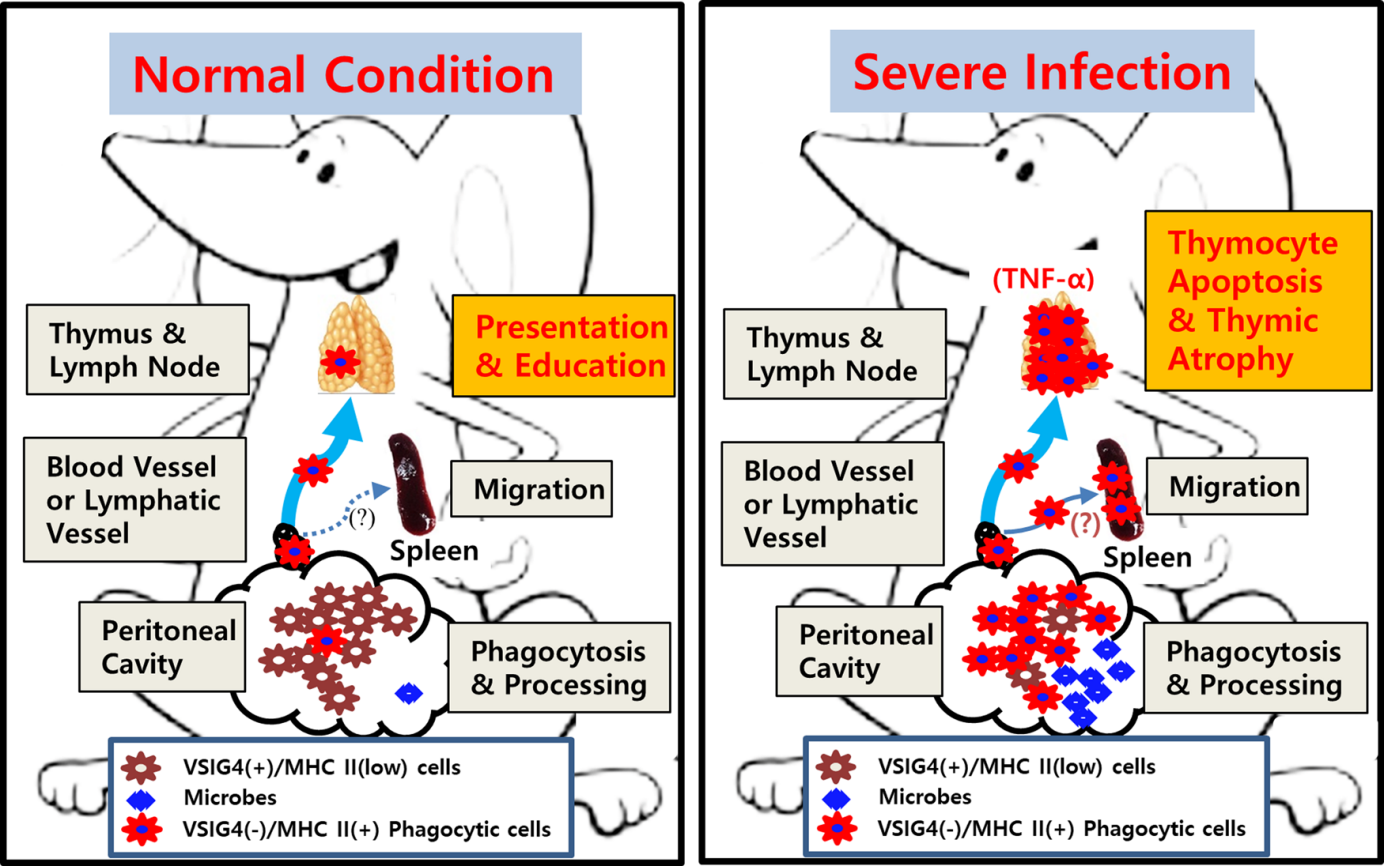
Schematic representation of the proposed mechanism for thymic atrophy in CLP Mice. Left panel: In normal condition, small peritoneal V4(+)/MHC II(low) cells ingest a small number of invading microbes and become activated cells. These activated cells migrate to thymus through blood or lymphatic vessels, and may change to V4(−)/MHC II(+) cells in process of migration. These activated V4(−)/MHC II(+) cells may be involved in the role of antigen-presenting cells in thymus. Right panel: In severe infection, many peritoneal V4(+)/MHC II(low) cells ingest a large number of invading microbes and become over-activated cells. Many over-activated cells vigorously migrate to thymus through blood or lymphatic vessels, and may change to V4(−)/MHC II(+) cells in process of migration. In thymus, “TNF-α” secreted by over-activated V4(−)/MHC II(+) cells induces apoptosis of DP thymocytes resulting in thymic atrophy.

Watts mentioned that MHC II molecules are limited in expression to professional APCs such as DCs, macrophages, B lymphocytes and are specialized to present antigens internalized from the extracellular space^23^. In our results, most V4(+) cells exhibited low expression of MHC II (Fig. 4a). As shown in Fig. 4b, these V4(+) cells also showed robust phagocytosis capacity compared to that of V4(−) cells. Furthermore, the migrated macrophages in the thymus after CLP induction exhibited a V4(−)/MHC II(+) phenotype (Figs. 3f, 4c). Based on these results, V4 (+)/MHC II (low) cells seem to function similarly to professional APCs under condition of infection. These findings also corresponded to previous reports showing that only resident macrophages in peritoneal lavage were positive for VSIG4 expression, and activated cells changed to a VSIG4-negative phenotype^14^. However, our results further indicate that under normal conditions, a small number of V4(−)/MHC II(+), which are believed to have been migrated from peritoneal cavity, found in the thymus or the mediastinal lymph node around the thymus. Therefore, V4(+)/MHC II(low) cells in the peritoneal cavity seem to be the preferred cells for antigen presentation after phagocytosis. In contrast, after activation, VSIG(−)/MHC II(+) cells seem to be mainly involved in antigen presentation and the removal of dead cells in thymus (Fig. 8, left). In conditions of severe infection, such as CLP, many V4(+)/MHC II(low) cells seem to migrate into the thymus after phagocytosis because of an increase in inflammatory cytokines and an uncontrolled inflammatory response, resulting in thymocyte apoptosis and thymic atrophy (Fig. 8, right).

TNF-α is well known as a proinflammatory cytokine and exerts its biological effects by signaling through its receptors TNFR-1 and TNFR-2. It is necessary for fetal thymocyte commitment, and for further thymocyte maturation and CD4^+^CD8^+^ differentiation^24^. Therefore, TNF-α is an essential cytokine for the survival of many cell types and also induces cell death at high concentrations^25^. Wang et al.^26^ demonstrated that intraperitoneal injection of Gram-negative bacteria induces thymic atrophy in mice and that the increased TNF-α in serum may be responsible for thymocyte apoptosis. However, the researchers did not know what kind of immune cells were involved in this process. In our study, when total peritoneal cells were cocultured with thymocytes, these cells did not significantly induce thymocyte apoptosis. To solve this problem, we separated V4(+) cells from total peritoneal cells. When purified V4(+) cells were added to the coculture system, we were able to see that apoptosis of DP thymocyte was greatly induced (Figs. 5a, b). Therefore, we next screened apoptotic factors using ELISA with various cytokines and antibodies, and identified the presence of TNF-α in the culture supernatant.

In summary, we have shown for the first time that in the CLP-induced mouse sepsis model, VSIG4-positive peritoneal macrophages actively migrate to the thymus and induce apoptosis in double-positive thymocytes, thereby resulting in thymic atrophy. These effects are thought to result in immunodeficiency not only in the disease progresses, but also after treatment. Thus, this is an important new finding in the field of sepsis, and controlling VSIG4-positive macrophages may be an effective method for preventing and treating immune paralysis by polymicrobial infection.

## Supplementary information

Supplementary Figure Legend

Supplementary Figure 1
