# Supplementary material for: VSIG4(+) peritoneal macrophages induce apoptosis of double-positive thymocyte via the secretion of TNF-α in a CLP-induced sepsis model resulting in thymic atrophy
Source: Cell Death Dis. 2021 May 22;12(6):526. doi: 10.1038/s41419-021-03806-5 (PMC8139869; doi:10.1038/s41419-021-03806-5)
Supplement: Supplementary file 1 — Supplementary Figure Legend [file 41419_2021_3806_MOESM1_ESM.docx]

**Supplementary Figure Legends**

**Fig. 1 Study using CLP-induced Wild-type and V4(KO) mice. a** V4(+) and V4(KO) macrophages were isolated from wild-type or V4(KO) mice using anti-VSIG4 or anti-CD11b antibody and MACS MicroBeads. Cells purity > 95% was obtained. Purified V4(+) and V4(KO) cells were stained with VivoTracker, and the stained cells were injected into the peritoneal cavities of normal mice after CLP induction. After 24 h, the mice were euthanized, and the peritoneal cavity was washed with PBS three times. Then, the fluorescence intensities of different organs were detected with an In Vivo Imaging System. **b** The thymus were isolated from mice and analyzed with an IVIS. **c** After IVIS analysis, thymocytes were purified and cells were stained with CD11b antibody, and then analyzed by flow cytometry. **e** The data indicates the percentage of CD11b/VTK cells. The data represent the mean ± S.E.M. of three separate experiments. **p<0.01, ANOVA. V4 indicates VSIG4, V4(+) indicates V4(+) cells, V4(KO) indicates V4(KO) cells, and VTK indicates the activity of VivoTracker.
