# Supplementary figures and images for: VSIG4(+) peritoneal macrophages induce apoptosis of double-positive thymocyte via the secretion of TNF-α in a CLP-induced sepsis model resulting in thymic atrophy
Source: Cell Death Dis. 2021 May 22;12(6):526. doi: 10.1038/s41419-021-03806-5 (PMC8139869; doi:10.1038/s41419-021-03806-5)

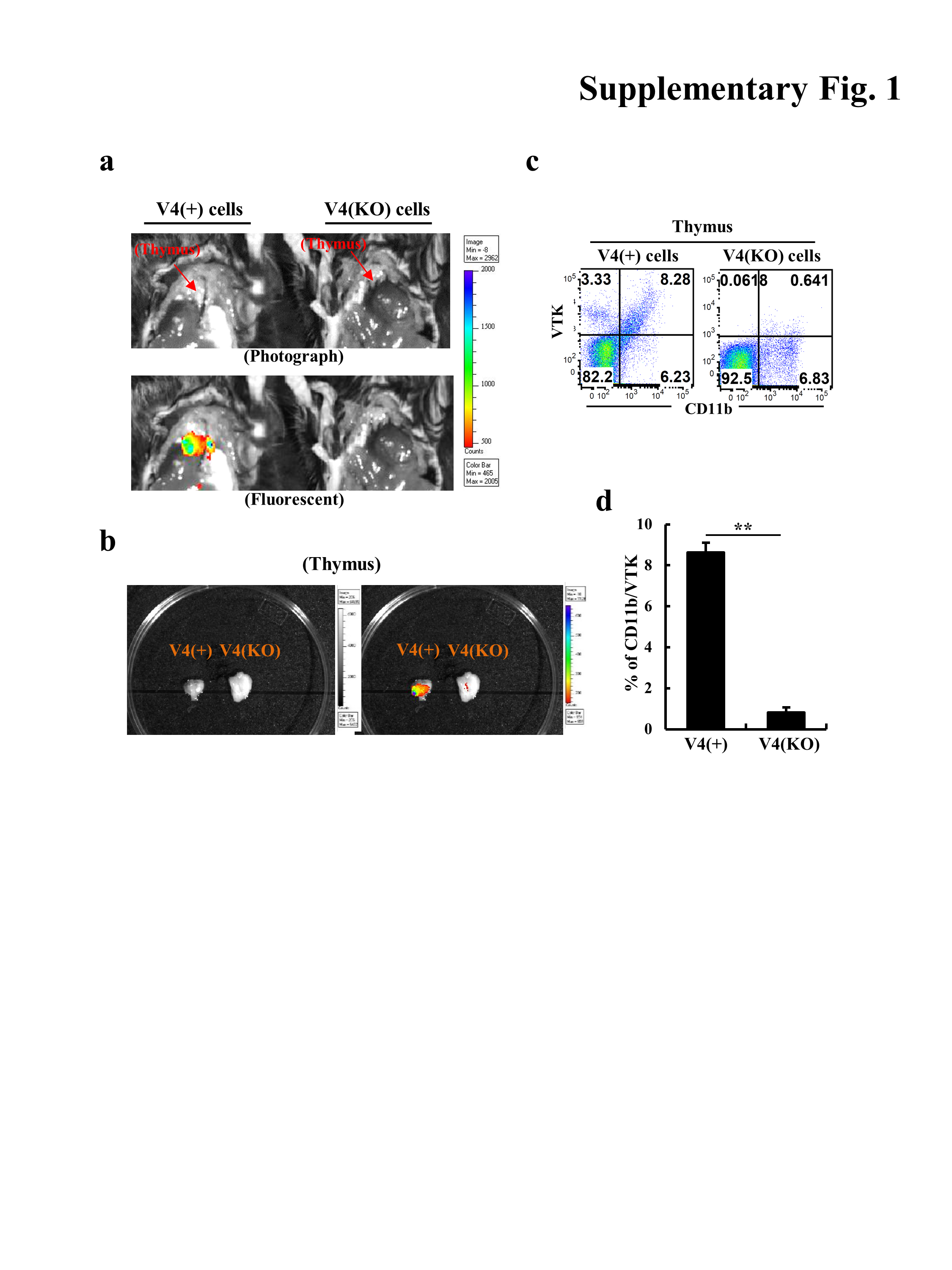

Supplement: Supplementary file 2 — Supplementary Figure 1 [file 41419_2021_3806_MOESM2_ESM.tif]
